# Nurr1, Pitx3, and α7 nAChRs mRNA Expression in Nigral Tissue of Rats with Pedunculopontine Neurotoxic Lesion

**DOI:** 10.3390/medicina55100616

**Published:** 2019-09-20

**Authors:** Lisette Blanco-Lezcano, Esteban Alberti-Amador, María Elena González-Fraguela, Guadalupe Zaldívar-Lelo de Larrea, Rosa Martha Pérez-Serrano, Nadia Angélica Jiménez-Luna, Teresa Serrano-Sánchez, Liliana Francis-Turner, Dianet Camejo-Rodriguez, Yamilé Vega-Hurtado

**Affiliations:** 1 International Center of Neurological Restoration (CIREN), Playa, Havana 10300, Cuba; alberti@neuro.ciren.cu (E.A.-A.); marie@neuro.ciren.cu (M.E.G.-F.); teresa@neuro.ciren.cu (T.S.-S.); dianetcr7@nauta.cu (D.C.-R.); yvega@neuro.ciren.cu (Y.V.-H.); 2 Faculty of Medicine, Autonomous University of Queretaro, Querétaro 76176, Mexico; apizl@yahoo.com.mx (G.Z.-L.d.L.); rositaperezserrano@gmail.com (R.M.P.-S.); nadia.jimenez@uaq.mx (N.A.J.-L.); 3 Experimental Group: “Experimental Models for Zoo-Human Sciences”, Faculty of Sciences, Tolima University, Ibagué 730001, Colombia; lilycolcuba@gmail.com

**Keywords:** pedunculopontine nucleus, nigral tissue, Nurr1, Pitx3, α7 nAChRs, mRNA expression

## Abstract

*Background and Objectives*: The knowledge that the cholinergic neurons from pedunculopontine nucleus (PPN) are vulnerable to the degeneration in early stages of the Parkinson disease progression has opened new perspectives to the development of experimental model focused in pontine lesions that could increase the risk of nigral degeneration. In this context it is known that PPN lesioned rats exhibit early changes in the gene expression of proteins responsible for dopaminergic homeostasis. At the same time, it is known that nicotinic cholinergic receptors (nAChRs) mediate the excitatory influence of pontine-nigral projection. However, the effect of PPN injury on the expression of transcription factors that modulate dopaminergic neurotransmission in the adult brain as well as the α7 nAChRs gene expression has not been studied. The main objective of the present work was the study of the effects of the unilateral neurotoxic lesion of PPN in nuclear receptor-related factor 1 (Nurr1), paired-like homeodomain transcription factor 3 (Pitx3), and α7 nAChRs mRNA expression in nigral tissue. *Materials and Methods*: The molecular biology studies were performed by means of RT-PCR. The following experimental groups were organized: Non-treated rats, N-methyl-D-aspartate (NMDA)-lesioned rats, and Sham operated rats. Experimental subjects were sacrificed 24 h, 48 h and seven days after PPN lesion. *Results*: Nurr1 mRNA expression, showed a significant increase both 24 h (*p* < 0.001) and 48 h (*p* < 0.01) after PPN injury. Pitx3 mRNA expression evidenced a significant increase 24 h (*p* < 0.001) followed by a significant decrease 48 h and seven days after PPN lesion (*p* < 0.01). Finally, the α7 nAChRs nigral mRNA expression remained significantly diminished 24 h, 48 h (*p* < 0.001), and 7 days (*p* < 0.01) after PPN neurotoxic injury. *Conclusion*: Taking together these modifications could represent early warning signals and could be the preamble to nigral neurodegeneration events.

## 1. Introduction

The neurodegenerative process in Parkinson disease (PD) is not restrained to dopaminergic neurons from substantia nigra pars compacta (SNpc) [1,2]. Post-mortem studies have shown that other neuronal populations (such as the cholinergic neurons of pedunculopontine nucleus, PPN) from brainstem, are also vulnerable to degeneration [1,2].

The works published by the Braak group [3,4] demonstrated the presence of Lewy bodies in the PNN in the stage III according to the Braak stages (I–VI) that describe the progression of PD [4]. Since then other studies have provided morphological and imagenological evidences that suggest an early commitment of the PPN during the progression of PD [5,6]. It is known that the loss of cholinergic neuron profiles in the PPN has a significant negative correlation with the modified Hoehn and Yahr scale [5]. Additionally, other authors published that the midbrain and pontine T1 (T1 is a potential marker for gray matter loss in qMRI study-3T) decrease in patients diagnosed with PD in Hoehn and Yahr stage I and II [6].

From the experimental point of view, as early as in 1992, the literature referred that excitotoxic lesions of the PPN may have an important role in creating better animal models of PD than classic models obtained by direct administration of neurotoxins that destroy the nigral dopaminergic neurons [7]. Extensive lesion of the PPN was reported to induce dopamine neuronal loss in the SNpc of rats [8]. More recently, other groups published that selective lesion of PPN cholinergic neurons resulted in a significant loss of dopamine neurons in the SNpc of rats [9].

Literature indicates that the nigral electrical activity is modulated, between others neurotransmitter system, by cholinergic pontine-nigral signals mediated by nicotinic and muscarinic receptors that are expressed on the nigral dopaminergic cells [10,11]. It has also been pointed out that the modification of cholinergic synapses can increase the vulnerability of nigral dopaminergic neurons to oxidative events that activate cell death programs [12].

Our group has published the occurrence of oxidative stress events at the nigrostriatal level in rats with PPN unilateral neurotoxic injury [13]. Moreover, from the molecular point of view, our studies demonstrated changes in the mRNA expression of proteins responsible for the nigrostriatal dopaminergic homeostasis (tyrosine hydroxylase (TH), vesicular monoamines transporter (VMAT2), and dopamine transporter (DAT)) seven days after PPN lesion [14].

These evidences suggested the possibility that the nigral expression of other genes and transcription factors could also be modified following the unilateral neurotoxic injury of PPN. Nuclear receptor-related factor 1 (Nurr1) and paired-like homeodomain transcription factor 3 (Pitx3), in whose signaling cascades are the genes related to dopaminergic homeostasis, are known to be involved in the differentiation to a dopaminergic phenotype during development and also play a regulatory role of dopaminergic homeostasis in the adult brain [15,16,17,18]. The literature points out that the expression of Nurr1, an orphan member of the nuclear receptor superfamily, is closely related to dopaminergic signaling [17]. The decrease in striatal dopamine release induced by 6-hydroxydopamine (6-OHDA) generates an early increase in nigral neurons Nurr1 expression [19]. This result has suggested that extracellular dopamine levels modulate the Nurr1 nigral expression [19]. Other studies have suggested that oxidative stress can cause the translocation of Nurr1 from the nucleus to the cytoplasm, which suggests that this gene can function as an important redox sensor in the central nervous system [20].

In relation to Pitx3, this is a transcription factor, whose correct expression is essential in the survival and maintenance of dopaminergic neurons [17]. Pitx3 shows restricted constitutive expression to the dopaminergic neurons of the SNpc and the ventral tegmental area (VTA) [21]. Knock out mice for Pitx3 show a marked decrease in the expression of TH together with a significant decrease in the density of nigral dopaminergic neurons compared with the wild type [22,23].

On the other hand, the reciprocal interaction between the SNpc and the PPN has suggested that these neuronal populations are mutually dependent on each other for the maintenance of their homeostasis and survival [9]. This interaction drives through the pontine cholinergic influence on the dopaminergic neurons of the SNpc, which is mediated by muscarinic (mAChRs) and nicotinic cholinergic receptors (nAChRs) [24]. In the brain, the nAChRs are expressed and function at the synapse (both pre-and postsynaptically) as well as extrasynaptically, and participate in postsynaptic responses [25]. The nAChRs are members of the superfamily of pentameric ligand-gated ion channels [26] and are presented in various combinations of sub-units (heteropentamers) or as homopentamers formed exclusively by α7 subunits [27,28]. α7 nAChRs is characterized by their fast desensitization kinetics as well as by their unusually high Ca^2+^ permeability [29]. This fact suggests important roles for that receptor in modulation of neurotransmitter release, gene expression, neuroprotection, and neurotoxicity [29]. Post-mortem studies have shown that the PPN neurons, which exerts an excitatory influence on the nigral dopaminergic neurons, show clear signs of degeneration [1,2,30]. This finding has suggested that the excitatory inputs mediated by nAChRs expressed in nigral neurons, can contribute to the survival of dopaminergic neurons [24]. This effect can progressively disappear during PD progression [11]. This hypothesis proposes that the loss of the pontine-nigral excitatory influence may increase nigral vulnerability to neurodegeneration events [24]. However, while the literature has focused on the study of neuroprotection attributed to nicotine [31,32,33], the effect of PPN neurotoxic injury on the nAChRs nigral expression has been less studied.

This work represents a continuation of the results published by our group which point out that the PPN neurotoxic injury increases the risk of nigral dopaminergic neurons to neurodegeneration. Our main objectives in the present work was the study of the effect of the PPN neurotoxic injury on the Nurr1, Pitx3, and α7 nAChRs mRNA expression in nigral tissue. Nurr1 and Pitx3 are two transcription factors that participate in the maintenance of the dopaminergic phenotype in the adult brain, while α7 nAChRs is partly responsible for mediating the pontine-nigral excitatory influence [15,16,17,18,24].

## 2. Materials and Methods

### 2.1. Experimental Subjects

Male, Wistar rats weighing 200–350 g, from the Centre for the Production of Laboratory Animals (CENPALAB), Mayabeque, Cuba, were housed 5 per cage under a temperature of 22–24 °C, with a relative humidity of 60% ± 5% and a light-darkness cycle of 12–12 h. Water and food were provided ad libitum. Experiments were carried out in accordance with the Cuban Regulations for the Use of Laboratory Animals (CENPALAB 1997) and were approved by the Ethical Committee of the International Center for Neurological Restoration. The protocol that guided the research was analyzed and approved by the Research Ethics Committee of CIREN in May 2017: (Code: ET.5-2017(2)).

### 2.2. Surgical Procedure

The rats were anesthetized with chloral hydrate (480 mg/kg i.p.) and placed in a frame of stereotactic surgery (David Kopf Instruments, Tujunga, CA, USA). The details of the procedure followed in the surgery have already been published previously by our group [7,8]. Briefly: The neurotoxic lesion of the PPN was carried out by means of the injection of an N-methyl-D-aspartate (NMDA) (Sigma, St. Louis, MO, USA) solution (0.5 μL, 0.1 M) in the coordinates (mm) corresponding to the PPN, according to the Paxinos and Watson atlas (2007) [34] AP: −7.80 ML: 1.60 DV: −7.60. At the end of the surgical procedure, the rats remained in postoperative care until their complete recovery [13]. Sham lesion of the PPN (sham-operated group): The surgical procedure was the same as that followed for injection of the neurotoxin but in place of the neurotoxin the rats received 0.5 µL of physiological saline solution (PSS).

Seven experimental groups were organized (Table 1) according to the administration of neurotoxin or physiological saline solution and to the temporal windows in which the gene expression was studied. The rats were injured, assigned randomly to each experimental group and sacrificed according to the temporal window (24 h, 48 h, and seven days post lesion) of interest for the study of gene expression.

### 2.3. Molecular Biology Studies

Sample collection: The dissections of the tissues were carried out under stereoscopic microscope (Wild Heerbrugg Stereo Microscopes, Heerbrugg, Switzerland). The animals were sacrificed by decapitation, after being anesthetized with chloral hydrate (480 mg/kg i.p.). The first step was to separate both hemispheres. Next, in the ventral portion of the right hemisphere the mesencephalon was divided with a cut at the caudal border of median eminence perpendicular to the long axis of mesencephalon in order to dissect the nigral tissue as it has been previously described [35]. Although we attempted to dissect the compact part of the SNpc only, a minor portion of the adjacent tissue could also be included to the final sample. The tissues were always dissected by the same investigator and under the same conditions. Taking into account that the pontine-nigral projection in rats is fundamentally ipsilateral [36], the molecular studies were performed on the right nigrostriatal tissue, ipsilateral to the PPN lesion.

### 2.4. RT-PCR Analysis for Gene Expression of Proteins: Nurr1, Pitx3, α7 nAChRs

Transcription factors mRNA expression was studied at 24, 48 h, and seven days after the PPN neurotoxic lesion. The total RNA was extracted from the nigral tissue of 40 rats using TRIzol^®^ Reagent (Invitrogen, Carlsbad, CA, USA). The yield and the integrity of RNA were determined by spectrophotometrical measurement of A260. The first-strand cDNAs were synthesized using the Go Taq G2, Hot Star Reverse Transcription System (Promega Corporation, Madison, WI, USA) in accordance with the protocol manufacturer’s instructions. Table 2 shows the data concerning the sequence of each primer used for RT-PCR, the annealing temperature (according to the size of the primers previously designed) and the size of each gene product. Cycling conditions were the recommended according protocol manufacturer’s instructions: One cycle a 45 °C for 45 min; 40 cycles for 2 min and 30 s at 94 °C and extension temperature at 68 °C for 7 min).

PCR products were separated by 2% agarose/ethidium bromide gel electrophoresis and visualized under UV light. Each electrophoresis was performed twice, followed by a semi-quantification analysis.

For the semi-quantitative study, the free online program ImageJ (Version 1.44; Wayne Rasband, National Institute of Health, Bethesda, MD, USA; http://imagej.nih.gov/ij) was employed. The analysis was performed according to the published method [37]. The background activity of the target band was subtracted and then normalized using β-actin as a reference.

### 2.5. Data Processing and Statistical Analysis

The values were expressed as mean ± SEM. Normal distribution and homogeneity of variance of the data were analyzed applying the Kolmogorov-Smirnov and Levene tests, respectively.

The comparison between experimental groups of the Nurr1, Pitx3, and α7 nAChRs mRNA expression was carried out by a one-way analysis of variance (ANOVA) followed by a multiple range test of Duncan. The statistical analysis was carried out for each time post injury separately. For all analyses, a significance level of 0.05 was considered using the Statistica 8.0 software (StatSoft Ink, Tulsa, OK, USA) software.

## 3. Results

### The Neurotoxic Injury of PPN Modified the Nigral mRNA Expression of the Three Genes Studied

**Figure 1:**
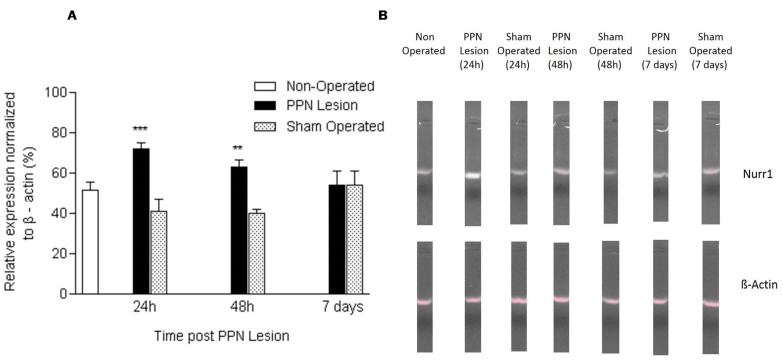
Effect of pedunculopontine nucleus (PPN) neurotoxic lesion on the nuclear receptor-related factor 1 (Nurr1) mRNA expression in nigral tissue. (**A**) Comparison between experimental groups 24 h (F (2, 14) = 13.29 *p* < 0.001), 48 h (F (2, 17) = 8.25 *p* < 0.01) and seven days (*p* > 0.05) after the lesion. The first bar (white bar) represents the non-operated group. This group is common for the ANOVA statistical analysis, which was carried out among non-operated, PPN lesion, and sham-operated groups for each time post lesion separately. The asterisks correspond to statistical differences between PPN lesion and both control groups (non-operated and sham operated). (**B**) Agarose/ethidium bromide gel electrophoresis bands representatives of the semi-quantitative RT-PCR study for Nurr1 mRNA expression. *** *p* < 0.001; ** *p* < 0.01.

Nurr1 mRNA expression showed an increase both at 24 h (F (2, 14) = 13.29 *p* < 0.001) and at 48 h (F (2, 17) = 8.25 *p* < 0.01) after PPN injury. Seven days after the lesion, no significant differences were found in the gene expression of Nurr1 between the experimental groups (*p* > 0.05) (Figure 1A,B). Figure 1.

**Figure 2:**
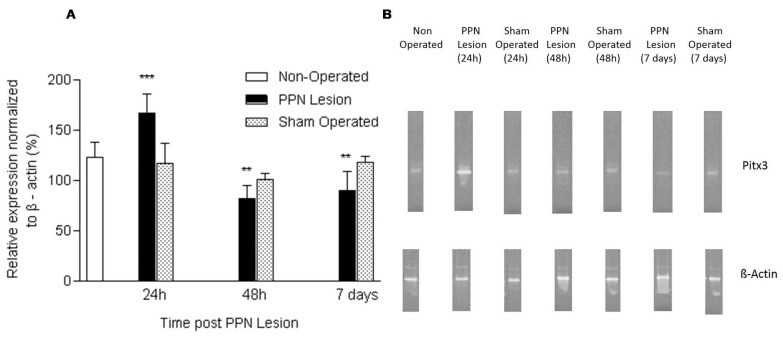
Effect of pedunculopontine nucleus (PPN) neurotoxic lesion on the paired-like homeodomain transcription factor 3 (Pitx3) mRNA expression in nigral tissue. (**A**) Comparison between experimental groups 24 h (F (2, 14) = 11.06 *p* < 0.001), 48 h (F (2, 17) = 7.22 *p* < 0.01) and seven days (F (2, 15) = 7.65 *p* < 0.01) after PPN lesion. The first bar (white bar) represents the non-operated group. This group is common for the ANOVA statistical analysis, which was carried out among non-operated, PPN lesion and sham-operated groups for each time post lesion separately. The asterisks correspond to statistical differences between PPN lesion and both control groups (non-operated and sham operated). (**B**) Agarose/ethidium bromide gel electrophoresis bands representatives of the semi-quantitative RT-PCR study for Pitx3 mRNA expression. *** *p* < 0.001; ** *p* < 0.01.

Pitx3 mRNA expression showed a significant increase 24 h after PPN injury (F (2, 14) = 11.06 *p* < 0.001). Following 48 h (F (2, 17) = 7.22 *p* < 0.01) and seven days (F (2, 15) = 7.65 *p* < 0.01) after the pontine lesion, the nigral Pitx3 mRNA expression showed a significant decrease in relation to the control groups (Figure 2A,B).

**Figure 3:**
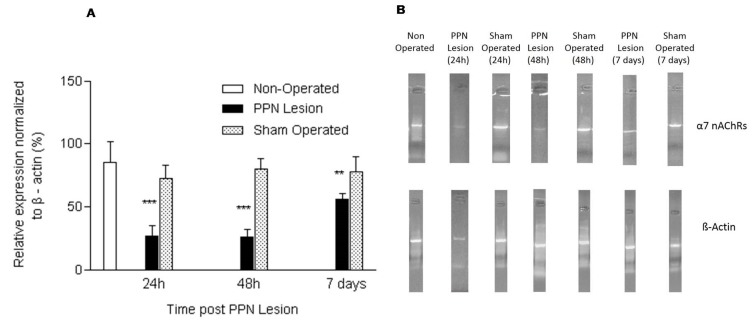
Effect of pedunculopontine nucleus (PPN) neurotoxic lesion on the nicotinic cholinergic receptors (α7 nAChRs) mRNA expression in nigral tissue. (**A**) Comparison between experimental groups 24 h (F (2, 14) = 10.31 *p* < 0.001), 48 h (F (2, 17) = 8.52 *p* < 0.001) and seven days (F (2, 15) = 7.12 *p* < 0.01) after the lesion. The first bar (white bar) represents the non-operated group. This group is common for the ANOVA statistical analysis, which was carried out among non-operated, PPN lesion and sham-operated groups for each time post lesion separately. The asterisks correspond to statistical differences between PPN lesion and both control groups (non-operated and sham operated). (**B**) Agarose/ethidium bromide gel electrophoresis bands representatives of the semi-quantitative RT-PCR study for α7 nAChRs mRNA expression. *** *p* < 0.001; ** *p* < 0.01.

Finally, the nigral α7 nAChRs mRNA expression remained significantly diminished 24 h (F (2, 14) = 10.31 *p* < 0.001), 48 h (F (2, 17) = 8.52 *p* < 0.001) and seven days (2, 15) = 7.12 *p* < 0.01) after the PPN neurotoxic injury in relation to the controls groups (Figure 3A,B).

In our previous paper, the histological verification of PPN lesion was already published because the molecular biology studies were carried out in the same samples [14]. Cresyl Violet study revealed the zone of injury in the distal part of the decussation of the superior cerebellar peduncle. The lesion does not appear to compromise this bundle of fibers [14]. At the same time, the inmunohistochemistry study revealed no significant differences between lesioned and control groups for the TH immunopositively neurons density in the SNpc seven days after PPN lesion [14].

## 4. Discussion

The main finding of this work is to demonstrate that the PPN neurotoxic injury induces transient, very early changes in the mRNA expression of transcription factors such as Nurr1 and Pitx3 related to the maintenance of the dopaminergic phenotype and dopaminergic neurotransmission. At the same time, the pontine lesion is accompanied by changes in the α7 nAChRs nigral expression, which had not been previously reported.

It is known that the expression of numerous genes is susceptible to modification when there are alterations in the homeostatic mechanisms that maintain cell survival. Our group has shown that the PPN neurotoxic injury is accompanied by a compromise of nigrostriatal oxidative homeostasis [13] as well as an increase in the brain derived neurotrophic factor (BDNF) mRNA expression in nigral tissue [38].

The literature reports that sublethal or prelethal oxidative stress can affect the trafficking of transcription factors between the nucleus and the cytoplasm of neurons and thereby modify the expression of these genes [39]. The very early transient increase in gene expression of Nurr1 and Pitx3 described in this work could be related to this scenario of oxidative stress that is characterized by an increase in catalase activity in nigral tissue early after PPN neurotoxic injury [13]. It is known that the changes associated with oxidative damage as well as the events that allow the restoration of cellular homeostasis can be accompanied by the activation or silencing of genes that encode antioxidant defense enzymes, transcription factors, carrier proteins, and structural proteins [40]. Other authors have described a nigral early over expression of different transcription factors following the injection of 6-OHDA into the striatum [41]. This over expression has been attributed an axoprotective role [41]. Accumulated data suggests that Nurr1 expression depends on dopamine signaling, essentially through D2 receptors [42]. D2 receptors are located in soma and dendrites, acting as autoreceptors that regulate the firing rate of neurons and presynaptically in nigrostriatal terminals to regulate dopamine synthesis and striatal release [11,43,44].

The PPN neurotoxic injury (through the injection of ibotenic acid) induces changes in the polysynaptic indirect basal ganglia pathway (which starts in striatal projection neurons that express dopaminergic receptors D2) [45]. These changes have been related to the hypoactivity of the nigral neurons as a consequence of a lower pontine—nigral excitatory influence after PPN lesion [45]. Although in the present study it was not possible to determine the release of striatal dopamine, our group recently published a significant decrease in the gene expression of VMAT2 and DAT in the nigrostriatal tissue seven days after PPN injury [14]. These results resemble the pre-motor stages of PD and suggest modifications in the dopaminergic control of the striatum that could underline the changes in Nurr1 mRNA expression in close association with the PPN neurotoxic lesion.

Additionally, the Nurr1 overexpression very early after PPN lesion could also play an axoprotective role for the dopaminergic terminals that originate in the SNpc and reach the PPN [46]. Nevertheless, other studies will be necessary to confirm this hypothesis.

Other authors have revealed a down regulation in Nurr1 nigral expression associated with the loss of nigral dopaminergic neurons that is evident in advanced stages of Parkinson’s disease [47]. Our work does not show contradictions with these results, taking into account that the approach we propose does not imply directly injuring the SNpc. Up to the temporal window that we studied in the present work (seven days), there is no evident morphological commitment in the density of TH neurons, according to our already published results [14].

In connection to Pitx3 mRNA expression, the literature has shown that Pitx3 interacts with the promoter of the TH gene improving its transcription in Neuro2A neuroblastoma cells [48]. The co-expression of Pitx3 and Nurr1 shows a synergistic effect in the stimulation of the TH promoter [48]. Our results showed a transient increased co-expression of Nurr1 and Pitx3 mRNA very early after PPN injury (24 h post injury) followed by a significant decrease in Pitx3 expression 48 h after the PPN lesion. The fact that the increased expression of both genes is not maintained over time could be responsible for not detecting a significant increase in the nigral expression of TH immediately after PPN injury [14]. However, the initial stimulus may be sufficient to facilitate the late expression of TH, which, according to our published results, is significantly higher than in controls, seven days after PPN injury [14].

In relation to the study of the expression of the α7 nAChRs, the finding of the decrease of this variable in rat nigral tissue very early after PPN injury represents an interesting result. Modifications in the functional availability of α7 nAChRs are reported in different neurodegenerative diseases [49]. The majority of the studies on nicotinic neural receptors address aspects related to the activation or desensitization of these in response to lower or higher concentrations of acetylcholine (ACh) or other agonists administered in pharmacological manipulation studies [50,51]. To our knowledge, this is the first study about the nigral expression of nAChRs in response to the unilateral neurotoxic injury of the PPN.

Acetylcholine plays a dominant role in shaping DA release probability and its dynamic short-term plasticity through action at nAChRs and mAChRs [52]. The nigral dopaminergic neurons express nAChRs that filter or modulate the cholinergic signal so that the dopaminergic neuron translates the cholinergic stimulation into release of striatal dopamine [27]. The filtering of the signal consists of a short-term depression of the synaptic nigrostriatal transmission that under physiological conditions limits the sub sequential release of dopamine (establishes a small pause). This physiological pause allows an optimal translation of action potential in dopamine release, improving the gain of the nigrostriatal dopaminergic signal and therefore the dopaminergic control over the striatum [27].

The PPN neurotoxic lesion compromises the pontine-nigral projection that is excitatory and mediated by acetylcholine and glutamate [53,54]. Previous studies point out that the pontine-nigral excitatory glutamatergic inputs are an additional excitatory source to dopamine neurons [53] due to it being well established in the literature, the existence of a subthalamic-nigral excitatory glutamatergic projection [55,56]. Other authors have suggested that this projection could compensate a possible decrease of the glutamatergic pontine-nigral inputs and at the same time, maintain an excitatory tone over nigral neurons in PPN experimental lesion [57,58]. In contraposition, morphological, and functional studies have revealed that nigral neurons receive an exclusive cholinergic projection from PPN [59,60,61]. Taken together these evidences could justify that the study of the pontine degeneration effects has been focused essentially (in the literature and in the present work) in the loss of the cholinergic component of the pontine-nigral inputs.

Possibly the transient decrease in the expression of the nigral nAChRs represents an early temporary warning signal in the SNpc associated with pontine hypoactivity. The nAChRs are involved in the phasic effects of acetylcholine under conditions of brief release/high local concentration of the neurotransmitter, but they also operate under the low ACh release [28]. The subsequent slow recovery of the α7 nAChRs expression could be consistent with the management of the receptor under a regime of lower release of ACh by the pontine-nigral terminal. Likewise, a possible role of oxidative stress by modifying the expression of α7 nAChRs cannot be ruled out. The literature reports that oxidative stress may contribute to the inactivation of α4β2-containing and α6β2-containing nAChRs in nigral dopaminergic neurons in the pre-symptomatic stage of PD [62]. However, other studies will be necessary to confirm this hypothesis.

In nigral neurons several subtypes of nicotinic cholinergic receptors are expressed, the α7 nAChRs represent approximately 40% of them [63]. α7 nAChRs contribute to activate a survival pathway involving the calcium effector protein calmodulin and phosphatidylinositol 3-kinase [64] as well as to attenuate the glial activation in 6-OHDA model [65]. These mechanisms mediated by nicotinic cholinergic receptors could contribute to delay the appearance of loss of nigral dopaminergic neurons since it is known that at least until the first seven days following the neurotoxic injury of the PPN no morphological compromise was observed in the nigral neurons [14].

Additionally, it is important to note that the changes in the nigral gene expression shown in the present work have been studied in the right SNpc, ipsilateral to the pontine lesion. Future studies should address the evaluation of these changes in the contralateral hemisphere, to elucidate the possible bilateral effects of the PPN neurotoxic lesion in these molecular events.

## 5. Conclusions

The neurotoxic injury of the PPN is part of the current approaches to an experimental model of PD that mimics the progressive course of this disease by increasing the nigral vulnerability to neurodegeneration events without directly damaging the SNpc. The pontine lesion induces very early changes in the gene expression of transcription factors that downstream participate in the expression of proteins and enzymes that guarantee the maintenance of dopaminergic homeostasis. These changes are concomitant with a scenario of nigrostriatal oxidative stress and a transient decrease in the expression of α7 nAChRs. Taking together, these modifications could represent early warning signals and could be the preamble to nigral neurodegeneration events.

## Figures and Tables

**Table 1:** Number of experimental subjects in each post-surgical time in which the gene expression was studied.

Experimental Groups	NMDA Injection	Sham-Operated Rats (Saline Injection)	Non-Operated Rats (Healthy Control Group)
			*n* = 6
Time post lesion			
24 h	*n* = 5	*n* = 4	
48 h	*n* = 6	*n* = 6	
7 days	*n* = 5	*n* = 5	

N-methyl-D-aspartate (NMDA).

**Table 2:** Sequence of primers for RT-PCR.

Gene Product	Sequence of Primers	Product Length (bp)	Annealing Temperature (°C)
Nurr1	5′ CGC CTT TCA CTC TTC TCC TTT A-3´	481	59
	5´ CTT CTT TAA CCA TCC CAA CAG C- 3´		
Pitx3	5´ GAG CAC AGT GAC TCG GAG AAG-3´	462	58
	5´ CGG TGA GAA TAC AGG TTG TGA A-3´		
α7 nAChRs	5´ AAA ATG CCT AAG TGG ACC AGA A-3´	401	58
	5´ TCA CTG CAG ATC ACC TCA CTC T-3´		
β-actin (endogenous control)	5´ ATT TGG CAC CAC ACT TTC TAC A-3´	379	60
	5´ TCA CGC ACG ATT TCC CTC TCA G-3´		
